# Use of Information and Communication Technologies Among Older People With and Without Frailty: A Population-Based Survey

**DOI:** 10.2196/jmir.5507

**Published:** 2017-02-14

**Authors:** Niina Susanna Keränen, Maarit Kangas, Milla Immonen, Heidi Similä, Heidi Enwald, Raija Korpelainen, Timo Jämsä

**Affiliations:** ^1^ Research Unit of Medical Imaging, Physics and Technology University of Oulu Oulu Finland; ^2^ Infotech Oulu Oulu Finland; ^3^ Medical Research Center Oulu Oulu University Hospital and University of Oulu Oulu Finland; ^4^ Center for Life Course Health Research University of Oulu Oulu Finland; ^5^ VTT Technical Research Centre of Finland Ltd Oulu Finland; ^6^ Information Studies Faculty of Humanities University of Oulu Oulu Finland; ^7^ Department of Sports and Exercise Medicine Oulu Deaconess Institute Oulu Finland; ^8^ Department of Diagnostic Radiology Oulu University Hospital Oulu Finland

**Keywords:** frail elderly, Internet, smartphone, population characteristics, logistic regression, aged

## Abstract

**Background:**

Use of information and communication technologies (ICT) among seniors is increasing; however, studies on the use of ICT by seniors at the highest risk of health impairment are lacking. Frail and prefrail seniors are a group that would likely benefit from preventive nutrition and exercise interventions, both of which can take advantage of ICT.

**Objective:**

The objective of the study was to quantify the differences in ICT use, attitudes, and reasons for nonuse among physically frail, prefrail, and nonfrail home-dwelling seniors.

**Methods:**

This was a population-based questionnaire study on people aged 65-98 years living in Northern Finland. A total of 794 eligible individuals responded out of a contacted random sample of 1500.

**Results:**

In this study, 29.8% (237/794) of the respondents were classified as frail or prefrail. The ICT use of frail persons was lower than that of the nonfrail ones. In multivariable logistic regression analysis, age and education level were associated with both the use of Internet and advanced mobile ICT such as smartphones or tablets. Controlling for age and education, frailty or prefrailty was independently related to the nonuse of advanced mobile ICT (odds ratio, OR=0.61, *P*=.01), and frailty with use of the Internet (OR=0.45, *P*=.03). The frail or prefrail ICT nonusers also held the most negative opinions on the usefulness or usability of mobile ICT. When opinion variables were included in the model, frailty status remained a significant predictor of ICT use.

**Conclusions:**

Physical frailty status is associated with older peoples’ ICT use independent of age, education, and opinions on ICT use. This should be taken into consideration when designing preventive and assistive technologies and interventions for older people at risk of health impairment.

## Introduction

The growing role of information and communication technologies (ICT) in our daily lives has led to concerns of increasing inequality between those who can and those who cannot take advantage of new technologies. The divide has been observed not only between younger and older people [1-3], but also within subgroups of older adults [2,4]. This is not only due to lack of access (first-level digital divide) but also due to lack of use (second-level digital divide) [4]. Advanced age, low education, low income, and disability have been shown to predict low Internet use among seniors [2,4-6].

ICT use can also be beneficial for older adults. Use of ICT for direct interaction with people or indirectly for other tasks has also been shown to contribute to the well-being and quality of life of older persons [7] and has been suggested to be associated with social engagement [7,8]. Moreover, ICT use offers a cognitively and intellectually challenging activity that can empower individuals [9]. Computers and Internet also provide many services that support autonomy in old age by facilitating the execution of many routine tasks through e-services (eg, banking, shopping, and communication with social and health services). Older adults who possess better cognitive skills are much better positioned to benefit from the Web-based services available [10].

The question remains, however, how greatly the digital divide among older adults hampers the potential reach of beneficial e-services such as eHealth within the groups that would most benefit from them. Previous studies on the ICT use of older adults with impaired health have used rather crude markers such as existing disability or a medical condition [2,11,12], or self-rated health [12,13]. In all these studies, the metric of poor health has predicted less ICT use, but the metrics of health may not highlight the ICT use and needs of home-dwelling older people most at risk of health impairment, hospitalization, and mortality.

Frailty, as a medical concept, is the loosely defined collection of physiological changes that results in an increased risk of adverse effects in response to stressors. Frail persons are at an elevated risk of falls, declining mobility or disability in daily activities, hospitalization, and death. For a frail person, any minor infection, trauma, or other events can cause a major change in health status and result in loss of independency [14,15]. Demographic factors that have been shown to be associated with frailty largely overlap with factors associated with low ICT use, including advanced age [14,16,17], low education [14,16,17], low income [14,17], and female gender [14,16]. Estimations of the prevalence of frailty vary, but are generally in the region of 10% in the community-dwelling older population [18].

In addition to the general benefits of ICT use for seniors, it could be utilized in various ways in the care of frail seniors in particular. Nutrition such as caloric and protein support [19] and physical activity such as personalized exercise [20,21] have been shown to have an effect on the progression of frailty. These are also potential targets for ICT-assisted interventions [22-25]. Frail older people are also active users of health and social services, yet many of them are deemed by frail users as inflexible, not catering to their needs, or impeded by the person’s limited mobility [26]. Frail individuals would benefit from individualized home-delivered health programs [27], which could also be digitally delivered. Mobile devices might also be used for the evaluation of frailty [28]. Additionally, ICT can be used for remote fall detection and rehabilitation among frail seniors [29,30].

In this population-based study, we aimed to compare the frail, prefrail, and nonfrail senior citizens regarding (1) ICT use, defined as use of Internet or advanced mobile devices (smartphones and tablet computers) and (2) attitudes toward the use of advanced mobile devices. We hypothesized that there would be lower ICT use among the frail seniors, and that the difference in ICT use would not be explained by age and education alone. Our aim was to produce new knowledge that can be utilized when developing effective interventions for persons at risk of impaired health and loss of independence.

## Methods

### The GASEL Survey

The study was conducted from November 2014 to January 2015 in Oulu. Oulu is Finland’s fifth largest city with a population of approximately 200,000, and 14% of the population is aged 65 years or more [31]. The study questionnaire was piloted among 11 volunteer seniors, and minor usability-related changes were made based on user feedback. A random sample of 1500 people living in Northern Finland was obtained from the Population Register Centre of Finland. The sampling criteria were as follows: (1) born during January 1, 1900 to December 31, 1949 (aged 65-114 years by the end of 2014); (2) spoke Finnish as a native language; and (3) had a permanent living address in Oulu, Finland, in November 2014. A reminder and another copy of the questionnaire were sent to the nonresponders 4 weeks after the first survey.

The study protocol was approved by the Ethics Committee of Human Sciences in the University of Oulu (statement 6/2014). Questions on ICT use, health, lifestyle, and sociodemographic factors were posed as described in the following sections. The study was part of the GASEL (Gamified Services for Elderly) project.

### Assessment of Frailty

Operational indicators of frailty are usually based on weight loss, fatigue, slowness, low physical activity, number of illnesses, and physical attributes such as low grip strength, inability to rise from a chair, or walk upstairs [19,32]. However, no single clear operational definition has been established [19].

To identify the frail study participants, we used a modified version of the 3-item SOF (Study of Osteoporotic Fractures) index, validated for the prediction of falls, disability, fracture, and mortality [32,33]. The modified items and rationale for modifications has been presented in Table 1. According to the responses, the responders were categorized as nonfrail (0 indicators), prefrail (1 indicator), or frail (2-3 indicators).

**Table 1:** The modified frailty index.

SOF^a^ index [13]	Modified item	Reason for modification
Weight loss of 5% in 3-4 years	At least 1 kg weight loss in 3 months	Easier to remember. Part of other scales used in the GASEL study.
Inability to rise from a chair 5 times without using arms	Inability to rise from a chair without using arms, as identified by “Can you rise from a chair independently and without using hands?” answered on a 5-point scale.	Risk of falling if the participants do the test unattended at home.
Poor energy as identified by “No” to “Do you feel full of energy?”	Poor energy as identified by “Which of the following best describes how energetic you have felt in the last month?” and response of “I feel moderately tired, exhausted or weak” or worse.	Fitting in with other scales. Adapted from the 15D instrument [34].

^a^SOF: Study of Osteoporotic Fractures.

### Use of the Internet

Internet use in the last 3 months was assessed with the question “How often on average did you use the Internet in the last 3 months?” with 3 frequency response options, and 1 “Not at all.” Internet access at home was assessed with the question “Do you or anyone in your household have access to the Internet at home? (by any device)” with the options “Yes,” “No,” and “I don’t know.” These questions are slightly modified for brevity from the ones used in Eurostat surveys [35], where Internet use or nonuse is a separate question.

### Use of Specific Devices Including Smartphones and Tablets

Use of specific devices was asked with the question “In the last 12 months have you used following devices” with the response options “Yes, without major difficulties,” “Yes, with difficulties,” “No,” and “I don't know.” The value of differentiating between use with difficulty and use without difficulty has been previously shown in the development of the everyday technology use questionnaire [36].

Smartphone use was asked with the question “Have you used a mobile phone with a touch display?” Although not all smartphones are touch phones or vice versa, this simplification made responding easier for the less technologically inclined.

Technology acceptance was asked based on the constructs of the Unified Theory of Acceptance and Use of Technology (UTAUT) [37]. Technology acceptance models strive to determine the variables, and the interactions of said variables, that predict the adoption of an available technology by an individual. UTAUT is part of a continuum of popular models that also includes the Technology Acceptance Model (TAM) [38], TAM2 [39], and UTAUT2 [40]. These models have been shown to be valid in a large number of contexts, countries, and population groups, and among older age groups [41].

UTAUT includes 3 constructs that predict behavioral intention to use the specific technology. Performance expectancy (PE) is defined as the degree to which technology provides benefits to users in performing certain activities; effort expectancy (EE) refers to the degree of ease associated with the use of technology; and social influence (SI) refers to the extent to which users perceive that important others (eg, family and friends) believe they should use a particular technology [40]. These determinants are moderated by gender, age, experience, and voluntariness of use. The determinants are usually assessed with a standard set of questions. An additional construct, facilitating conditions, refers to the degree to which an individual believes that an organizational and technical infrastructure exists to support the use of the system and affects the transition from intention to use [37].

We used UTAUT as a list of factors affecting technology adoption of mobile technologies such as smartphones or tablets. Facilitating conditions could not be assessed because of the many possible types and the length of the questionnaire. Outside of the questions rooted in the UTAUT framework, the questionnaire was supplemented with a general interest question, a question regarding cost, and a question about privacy concerns. Only 1 question per determinant was included and answered with “Yes,” “No,” or “I don’t know.” For regression analyses, “No or I don’t know” was used as the reference category [42].

### Socioeconomic Characteristics

The type of habitation of the participants was assessed with the question “Which of the following best describes your current form of residence?,” with 3 options corresponding to single- and multistory buildings with or without elevator, and one “assisted living building, rehabilitation center, or nursing home.”

Economic situation was assessed with the question “Is your household income sufficient for your needs?” with the options of “Yes, very well”; “Yes, reasonably well”; “Barely”; and “No (I am using up my savings or being supported by my friends and family).” The latter 2 were classified in the analyses as financial concerns.

Sensory problems were assessed with the dichotomous questions “Do you have difficulties in close activities (eg, handcrafts, reading) due to poor vision?” and “Do you have difficulties hearing (eg, using the phone)?” with the guidance to answer according to experiences in daily life, using the assistive devices usually used.

Mobility outside of the house was assessed with a combination of 2 questions. The Mini Nutritional Assessment (MNA) embedded within the survey included the question “Which of the following best describes your current mobility?” The response options were “I am bed or chair bound”; “I am able to get out of bed/chair, but do not go out”; and “I am able to move outside the house.” This was supplemented with the question “Do you use mobility aids?” with a list of various mobility aids and the multiple options “Indoors,” “Outdoors,” and “Not at all.” The first 2 MNA response options were considered to indicate indoors only, with the exception of a “Bed or chair bound” person who used mobility aids outdoors.

Loneliness was assessed with the 6-item Gierveld scale, which scores emotional and social loneliness on a scale of 0-6 [43]. We used the questions and the 5-point answer scale for responses previously translated into Finnish for the 11-item long version [44], which includes the 6-item scale as a subset.

Medical conditions were asked with a dichotomous answer to a list of possible diagnosed conditions. Dementia was asked separately as “mild dementia” or “moderate or severe dementia.” In cases where a respondent had answered at least one medical condition question, it was assumed blank answers correspond to “No.”

Education was asked on a 4-level scale, with the options “Less than primary education”; “Primary education”; “High school or college”; and “University degree or similar.” High school or above was considered higher education.

Participation on organized activities outside of the home environment was asked with the question “Do you participate in the activities of a club, union, society, hobby group or spiritual or religious society (eg, sports group, political party, choir, congregation)?” with the answer options “Yes, frequently,” “Yes, occasionally,” and “No.”

### Statistical Analysis

The data of each respondent were included in this study if the respondent (1) answered to all the frailty index questions; or scored 2 points in the index (answer to the additional question would not have changed the classification); (2) did not have moderate or severe dementia; and (3) lived in an environment that was not an assisted living facility or nursing home.

Kendall tau was used for crosstabs with ordinal variables and all 3 responder groups. Chi square tests were used to analyze the statistical significance of the differences in dichotomous frailty categories (“nonfrail” and “frail or prefrail”; or “nonfrail or prefrail” and “frail”). Independent-samples Kruskal–Wallis test was used to evaluate the significance of differences in continuous variables (age, number of daily medications) between the groups.

Univariate associations between explanatory variables and ICT use were first analyzed using cross-tabulation. Factors associated with ICT use in univariate analyses were entered into the multivariable logistic regression analysis. Age was first entered into the model, followed by the significant variables using forward stepwise (likelihood ratio) method as a second block. Two models were built and tested, 1 for each of the 2 different dichotomous classifications of frailty.

All statistical analyses were performed using SPSS Statistics version 22, 64-bit edition (IBM). The results were presented as odds ratios (ORs) with 95% CIs. The statistical significance was set at *P* value <.05.

## Results

### Socioeconomic Characteristics of the Responder Groups

By January 2015, 918 responses had been received, resulting in a response rate of 61.2%. After checking against inclusion criteria, a total of 794 responses (52.9% of the original sample) were included in the analysis. The responders were approximately 2.5 years younger than nonresponders or those who were excluded (*P*<.001), but there was no statistically significant difference in gender distribution (*P*=.42). Fifty-six (7.1%) were classified as frail, 181 (22.8%) as prefrail, and 557 (70.2%) as nonfrail.

Age (*P*<.001), financial concerns (*P*<.001), number of daily medications (*P*<.001), prevalence of mild dementia (*P*<.001), and trouble with near vision (*P*<.001) or hearing (*P*=.001) were positively associated with frailty level, whereas mobility outside home (*P*<.001) and participation in organized activities such as clubs, societies, political parties, or church (*P*<.001) were inversely associated with frailty level (ordinal analyses where applicable). A summary of these findings is shown in Multimedia Appendix 1. There were also significant differences in the average loneliness scores of the different groups (nonfrail 2.05, prefrail 2.63, frail 3.39; *P*<.001).

### Use of the Internet

The use of ICT was different across the frailty groups. Frailty level was significantly and inversely associated with having Internet connection at home (78.6% of nonfrail, 70.2% of prefrail, 46.4% of frail, *P*<.001), Internet use in last 3 months (71.8% of nonfrail, 64.1% of prefrail, 33.9% of frail, *P*<.001), and computer use in the last 12 months (70.0% of nonfrail, 62.4% of prefrail, 30.4% of frail, *P*<.001).

In univariate analyses, age, higher education, prevalence of mild dementia, poor near vision, and dichotomous frailty status were associated with Internet use, whereas gender was not (Multimedia Appendix 2). The results of the multivariable logistic regression with these variables are shown in Table 2.

Among those participants who had used the Internet during the last 12 months (n=556), there were no statistically significant differences in the type of Internet use between the different frailty level groups. The most common types of Internet use were e-services such as bank, social services, taxes, and tickets (86.2%); information such as timetables, health information, or recipes (83.3%); news (81.1%); communication such as email or Skype (70.1%); entertainment such as movies, books, and music (40.6%); and gaming (34.9%). Shopping, hobbies or studying, social media, and following or posting on forums were less frequent uses.

**Table 2:** Socioeconomic predictors of Internet use in the last 3 months among Finnish seniors aged 65+ years, according to multivariable regression analysis adjusted for age (n=738).

Socioeconomic predictors	Model 1^a^			Model 2^b^		
	Odds ratio	95% CI	*P* value	Odds ratio	95% CI	*P* value
Age (years)	0.87	0.84-0.90	<.001	0.87	0.84-0.90	<.001
High education	5.14	3.28-8.07	<.001	4.97	3.17-7.80	<.001
Poor near vision	0.65	0.45-0.94	.02	0.67	0.47-0.98	.04
Frailty				0.45	0.22-0.91	.03

^a^Nonfrail or prefrail is the reference category.

^b^Frail is the reference category.

### Use of Specific Devices Including Smartphones and Tablets

The majority of all responders, including the frail participants, used a regular mobile phone. Smartphones and tablets were used by less than a third of the responders and a small minority of frail persons, and the frail group experienced more difficulties in the use of all items. Only a small minority of responders in any group used a computer or mobile phone designed for seniors or other users with special needs (Multimedia Appendix 3).

In univariate analyses, higher education, prevalence of mild dementia, and dichotomous frailty levels were all associated with use of advanced mobile ICT (smartphones or tablets), but gender or poor near vision was not (Multimedia Appendix 4). The results of multivariable regression analysis with these variables are shown in Table 3. The frail people were not compared with the “prefrail or nonfrail” reference category due to the small number of subjects in the “frail, uses advanced mobile ICT” group (n=7).

When exploring opinions of advanced mobile ICT in ordinal analyses including all 3 response options, we found that nonusers had more negative opinions on performance expectancy (*P*<.001), interest (*P*<.001), effort expectancy (*P*=.002), social influence (*P*<.001), and subjective cost (*P*=.04). Differences in privacy concerns were not statistically significant between users and nonusers (*P*=.50). Not all of these differences can be seen in the univariate regression for dichotomized variables, which is presented in Multimedia Appendix 5. Multivariable regression using the UTAUT-derived variables performance expectancy, effort expectancy, social influence, as well as mild dementia and frailty can be seen in Table 4.

**Figure 1:**
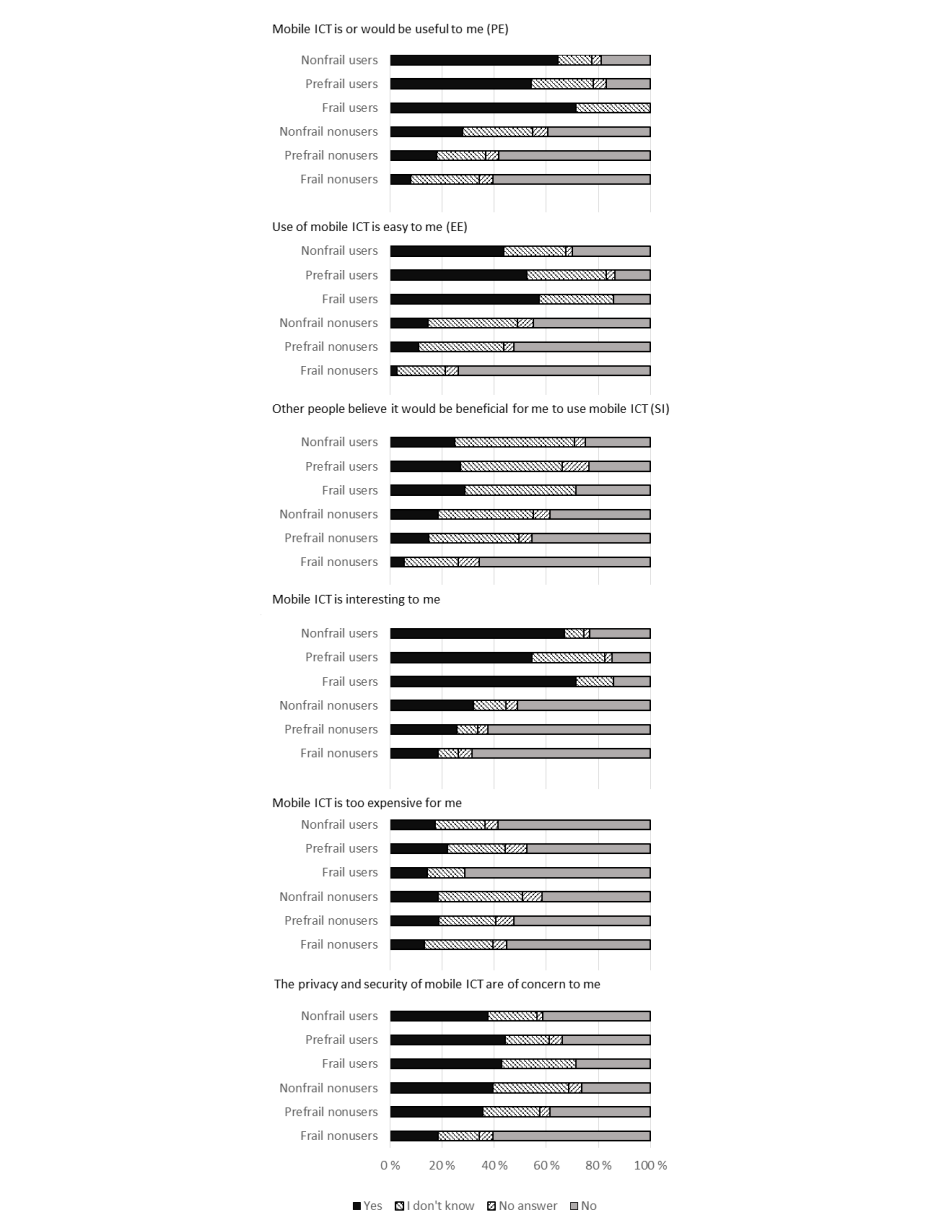
Potential factors on the adoption of advanced mobile ICT, including questions regarding UTAUT performance expectancy (PE), effort expectancy (EE), and social influence (SI). Users: n_nonfrail_=227; n_prefrail_=57; n_frail_=7. Nonusers: n_nonfrail_=254; n_prefrail_=101; n_frail_=38.

The frail nonusers in particular were more negative than nonfrail nonusers on opinion scales (Figure 1). Among nonusers, ordinal frailty status was significantly correlated with performance expectancy (*P*<.001), effort expectancy (*P*=.002), social influence (*P*=.003), privacy concerns (*P*=.001), and interest (*P*=.01), but not with subjective cost (*P*=.07). Among users, there were no such differences; and frailty status was positively correlated with effort expectancy (*P*=.007). However, the number of frail users of advanced mobile ICT was very low.

**Table 3:** Socioeconomic predictors of the use of advanced mobile information and communication technologies (ICT) in the last 12 months among Finnish seniors aged 65+ years, according to multivariable regression analysis adjusted for age (n=664).

Socioeconomic predictors	Odds ratio	95% CI	*P* value
Age (years)	0.92	0.89-0.95	<.001
High education	2.64	1.89-3.69	<.001
Frailty or prefrailty	0.61	0.42-0.89	.01

**Table 4:** Attitude predictors of the use of advanced mobile information and communication technologies (ICT) in the last 12 months among Finnish seniors aged 65+ years, according to multivariable regression analysis adjusted for age and education level (n=626).

Attitude predictors	Odds ratio	95% CI	*P* value
Age (years)	0.95	0.92-0.98	.002
High education	1.90	1.31-2.77	.001
Performance expectancy^a^	2.56	1.70-3.98	<.001
Effort expectancy^a^	2.39	1.48-3.86	.001
Frailty or prefrailty	0.60	0.40-0.92	.02

^a^Reference category is those who answered “No” or “I don’t know.”

## Discussion

### Principal Findings

Frail seniors represent an important target group for ICT-assisted interventions for the elderly. In this study, we, for the first time, compared the ICT use between frail and nonfrail older people at the population level. The observed lesser ICT use among the frail or prefrail people was not explained by age, education, memory disorders, visual impairments, or opinions on performance or effort to use.

Frailty and low ICT use share risk factors. In this study, the association of Internet use and frailty was only partially explained by the different demographics. The types of Internet use between the frail and nonfrail people were similar. The association of lower use of advanced mobile ICT and frailty was not explained by demographics or attitudes toward the technology.

In general, the frail nonusers were more negative toward advanced mobile ICT than the nonfrail nonusers; there was no such difference in attitudes among users. While the frail respondents reported more difficulties with making ends meet financially, cost was not rated as an obstacle to ICT use among frail nonusers.

### Comparison With Prior Work

Our findings are in agreement with previous research results on the prevalence of frailty and general ICT use of the senior population. The prevalence of frailty in this study (7.1%) is similar to previous estimates of 8.6-12.4% of community-dwelling people aged more than 65 years in northern Europe [16]. Here, 67.4% of older people had used the Internet in the last 3 months, and 74.4% had Internet at home. According to Eurostat, 68% of Finnish individuals aged 65-74 years have used the Internet in the last 3 months [3] (2014), and 63% of the age group have Internet access at home [45] (2013). Furthermore, our findings on the demographics of frail persons in Oulu correspond to the previously known higher age, lower education, and lower income than the general senior population. No gender difference was observed. Based on these statistics, we can consider the sample to be representative.

Multiple previous studies have looked into the ICT use of seniors with impaired health, but using different definitions of health. The presence of disability has been shown to be associated with lower Internet use, having fast Internet access at home, less ownership of major digital devices, and less use of email or SMS messages [2,12,46]. Presence of medical conditions [46,47] and low self-rated health [12,13,46] are associated with lower Internet, email, or SMS use. As an addition to these works, frailty is an interesting health classifier. Disability may indicate a functional impairment that may impose restrictions on ICT use while affecting the likelihood of hospitalization or mortality to a much lesser degree, and our findings were not explained by the prevalence of visual impairment. Medical conditions are common among seniors, and approximately half have at least two comorbid conditions [14]; as such it does not alone identify the most high-risk population, whereas the frail or prefrail people represent approximately 10-20% of the age group. Self-rated health has been shown to reflect the risk of mortality and functional impairment [48], but also personality type [49]. It is possible that self-perceived poor health and lack of ICT use share common personality features and general self-perception that are not directly related to health. Physical frailty is also often associated with psychosocial changes [50], such as diminished perception of personal growth and environmental mastery [51], and social isolation and depression [52]; however, metrics of frailty do not rely on the subject’s own evaluation alone.

The original TAM model [38] preceding UTAUT used the constructs “perceived usefulness” and “perceived ease-of-use,” which roughly correspond to PE and EE in UTAUT. These factors have previously been shown to be of chief importance for the technology acceptance of older people [41], and were the most significant predictors of use in our study population as well. Other studies on the nonuse of the Internet by seniors have shown that lack of interest, skepticism of usefulness, and doubt of one’s own skills to use are common attitudes among older ICT nonusers [2,6,53]. We found these same attitudes with regard to advanced mobile ICT. All these negative attitudes were emphasized among frail nonusers, suggesting that enticing this population to use ICT-based services will be particularly difficult.

Our findings offer further evidence that the correlation between low ICT use and health impairment reflects health status and risk, and not simply disability, self-perception, or the personality of the respondent. While older people at large are increasingly using computers, mobile technologies, and the Internet, it is still difficult to reach those most in the need of health care support through such means. The observed similar attitudes among users of mobile ICT regardless of frailty status, as well as the similar uses of the Internet, suggest that there are barriers of entry, rather than different needs. Previous research has divided barriers of ICT adoption with regards to eHealth into (1) physical and psychological attributes; (2) provision; (3) support from others; and (4) economic barriers [53,54]. Based on our study, the difference between frail and nonfrail seniors’ ICT adoption is unlikely to be primarily physical or economic, as neither visual disability nor subjective cost was significant in the multivariable analyses. There are psychosocial features correlated with physical frailty [51,52], which were not included in our models and could discourage the persons from trying new technologies. Possible higher prevalence of social isolation and subsequent lack of recommendations or pressure from peers [4,54] or perceived general helplessness or powerlessness [54] are possible reasons, as are subclinical or undiagnosed cognitive impairments. These should be assessed in further studies targeting health and well-being differences in ICT use.

### Limitations

The response rate was high in this study; however, the survey was voluntary. It is likely that the frail are underrepresented in the sample, compared with the general home-dwelling senior population. In particular, the group of frail seniors using advanced mobile ICT was very small, which limited the statistical analyses.

We modified the UTAUT survey for the purposes of this study. The UTAUT model has been built for use with specific use cases rather than broad categories such as “mobile ICT,” and we did not use the standard question set. Thus, the results should only be used for the context of this paper, not as validation of the UTAUT model for this purpose.

Due to the constraints of the survey format and the large number of questions, there were some limitations in the depth of assessment. In particular, controlling for more detailed assessments of cognitive functioning, depressive symptoms, and social network would have been interesting, and should be taken into account in future studies.

### Conclusions

This study provides early evidence that community-dwelling nonfrail and frail older people are highly different as ICT users, both in actual use rate and in opinions affecting adoption among the nonusing population. These differences are only partly explained by more advanced age, lower education, and higher prevalence of visual or cognitive impairments. As such the research also highlights the clustering of various psychological and social risk factors of social exclusion to the same physically vulnerable population, and the continuing need of measures to prevent an increasing digital divide in society. Future research is needed to further assess the ICT-related needs, barriers to adoption, and facilitating conditions of this interesting and important population.

## Appendix

Multimedia Appendix 1 Characteristics of the study participants according to frailty status.

Multimedia Appendix 2 Socioeconomic predictors of Internet use in the last 3 months among Finnish seniors aged 65+ years in univariate regression analysis.

Multimedia Appendix 3 Information and communication technologies (ICT) device use during the last 12 months and difficulties among users across the frailty categories.

Multimedia Appendix 4 Socioeconomic predictors of the use of advanced mobile information and communication technologies (ICT; smartphones or tablets) in the last 12 months among Finnish seniors aged 65+ years in univariate regression analysis.

Multimedia Appendix 5 Attitude predictors of the use of advanced mobile information and communication technologies (ICT) in the last 12 months among Finnish seniors aged 65+ years in univariate regression. “I don’t know” answers counted as negative.

## Abbreviations

EE: effort expectancy
FAC: facilitating conditions
ICT: information and communication technologies
PE: performance expectancy
SI: social influence
UTAUT: Unified Theory of Acceptance and Use of Technology
SOF: Study of Osteoporotic Fractures
