# Supplementary material for: Use of Information and Communication Technologies Among Older People With and Without Frailty: A Population-Based Survey
Source: J Med Internet Res. 2017 Feb 14;19(2):e29. doi: 10.2196/jmir.5507 (PMC5331186; doi:10.2196/jmir.5507)
Supplement: Multimedia Appendix 1 [file jmir_v19i2e29_app1.pdf]

Multimedia Appendix 1. Characteristics of the study participants according to frailty status.

| Socioeconomic characteristics | Nonfrail | Prefrail | Frail | *P* |
|---|---|---|---|---|
| % of responses | 70.2 | 22.8 | 7.1 | |
| | N=557 | N=181 | N=56 | |
| Age in years, mean (SD) | 72.0 (6.0) | 73.8 (6.9) | 77.4 (7.4) | <.001[a] |
| Aged 85 years or more, % (n) | 3.8 (21) | 7.7 (14) | 17.9 (10) | .002[b] |
| Female, % (n) | 56.9 (317) | 56.4 (102) | 55.4 (31) | .82[b] |
| Mild dementia, % (n) | 8.1 (45) | 16.0 (29) | 32.1 (18) | <.001[b] |
| | N=553 | N=178 | N=55 | |
| Education, high school or above, % (n) | 36.7 (203) | 42.1 (75) | 16.4 (9) | .48[b] .10[c] |
| | N=547 | N=169 | N=49 | |
| Moves outside of house, % (n) | 100.0 (547) | 92.3 (156) | 71.4 (35) | <.001[b] |
| | N=513 | N=175 | N=50 | |
| Financial concerns, % (n) | 8.6 (44) | 21.7 (38) | 28.0 (14) | <.001[bc] |
| | N=316 | N=113 | N=28 | |
| Medication, 6 or more daily, % (n) | 6.0 (19) | 25.7 (29) | 57.1 (16) | <.001[ab] |
| | N=541 | N=176 | N=52 | |
| Trouble with near vision, % (n) | 37.0 (200) | 48.9 (86) | 59.6 (31) | <.001[b] |
| | N=531 | N=171 | N=52 | |
| Trouble with hearing, % (n) | 14.9 (79) | 21.1 (36) | 36.5 (19) | .001[b] |
| | N=537 | N=174 | N=53 | |
| Participates in organized activities, % (n) | 65.9 (354) | 56.3 (98) | 49.1 (26) | .003[b] <.001[c] |

[a]Kruskal–Wallis, continuous variable (age, number of medications).

[b]Kendall tau, 2 categories across frailty groups.

[c]Kendall tau, multiple ordinal categories across frailty groups.
