# Supplementary material for: Use of Information and Communication Technologies Among Older People With and Without Frailty: A Population-Based Survey
Source: J Med Internet Res. 2017 Feb 14;19(2):e29. doi: 10.2196/jmir.5507 (PMC5331186; doi:10.2196/jmir.5507)
Supplement: Multimedia Appendix 2 [file jmir_v19i2e29_app2.pdf]

Multimedia Appendix 2. Socioeconomic predictors of Internet use in the last 3 months among Finnish seniors aged 65+ years in univariate regression analysis.

| Socioeconomic predictors | Internet user | Internet nonuser | Odds ratio | 95% CI | *P* |
|---|---|---|---|---|---|
| % of responses | 69.9 | 30.1 | | | |
| | N=535 | N=230 | | | |
| Mean age (SD) | 70.8 (5.2) | 76.7 (7.0) | 0.86 | 0.83-0.88 | <.001 |
| Woman, % (n) | 54.6 (292) | 60.9 (140) | 0.77 | 0.56-1.1 | .11 |
| Mild dementia, % (n) | 6.7 (36) | 21.7 (50) | 0.26 | 0.16-0.41 | <.001 |
| Frailty or pre-frailty, % (n) | 25.2 (135) | 39.1 (90) | 0.53 | 0.38-0.73 | <.001 |
| Frailty, % (n) | 3.6 (19) | 13.5 (31) | 0.24 | 0.13-0.43 | <.001 |
| | N=529 | N=229 | | | |
| High education, % (n) | 47.8 (253) | 13.5 (31) | 5.86 | 3.87-8.97 | <.001 |
| | N=522 | N=222 | | | |
| Poor near vision, % (n) | 37.0 (193) | 49.5 (110) | 0.60 | 0.44-0.82 | .001 |
