# Supplementary material for: Use of Information and Communication Technologies Among Older People With and Without Frailty: A Population-Based Survey
Source: J Med Internet Res. 2017 Feb 14;19(2):e29. doi: 10.2196/jmir.5507 (PMC5331186; doi:10.2196/jmir.5507)
Supplement: Multimedia Appendix 3 [file jmir_v19i2e29_app3.pdf]

Multimedia Appendix 3. Information and communication technologies (ICT) device use during the last 12 months and difficulties among users across the frailty categories.

Device use, % (n)	Nonfrail	Prefrail	Frail	*P*
	N=527	N=168	N=50	
Mobile phone	96.4 (508)	93.5 (157)	84.0 (42)	0.01
Difficulties in using mobile phone % of users (n)	2.4 (12)	3.2 (5)	4.8 (2)	.41
	N=499	N=165	N=46	
Computer	78.2 (390)	68.5 (113)	37.0 (17)	<.001
Difficulties in using computer % of users (n)	11.5 (45)	17.7 (20)	23.5 (4)	.06
	N=457	N=155	N=42	
Tablet	29.5 (135)	21.9 (34)	7.1 (3)	.001
Difficulties in using tablet % of users (n)	9.6 (13)	14.7 (5)	33.3 (1)	.30
	N=459	N=149	N=44	
Smartphone[a]	37.0 (170)	28.9 (43)	11.4 (5)	<.001
Difficulties in using smartphone % of users (n)	4.1 (7)	7.0 (3)	20.0 (1)	.31
	N=456	N=155	N=45	
Senior ICT[b]	2.9 (13)	5.8 (9)	4.4 (2)	.155

[a]Defined as any mobile phone with a touch display.

[b]Computer or mobile phone designed for seniors or people with physical impairments. ICT: information and communication technologies.
