# Supplementary material for: Use of Information and Communication Technologies Among Older People With and Without Frailty: A Population-Based Survey
Source: J Med Internet Res. 2017 Feb 14;19(2):e29. doi: 10.2196/jmir.5507 (PMC5331186; doi:10.2196/jmir.5507)
Supplement: Multimedia Appendix 4 [file jmir_v19i2e29_app4.pdf]

Multimedia Appendix 4. Socioeconomic predictors of the use of advanced mobile information and communication technologies (ICT; smartphones or tablets) in the last 12 months among Finnish seniors aged 65+ years in univariate regression analysis.

Socioeconomic predictors	Mobile ICT[a] user	Mobile ICT nonuser	Odds ratio	95% CI	*P*
% of responses	42.7	57.2			
	N=293	N=393			
Mean age (SD)	70.3 (5.0)	73.7 (6.8)	0.91	0.88-0.93	<.001
Woman, % (n)	53.6 (157)	60.1 (236)	0.77	0.57-1.04	.09
Mild dementia, % (n)	6.8 (20)	14.0 (55)	0.45	0.26-0.77	.004
Frailty or prefrailty, % (n)	22.5 (66)	35.4 (139)	0.53	0.38-0.75	<.001
Frailty, % (n)	2.4 (7)	9.7 (38)	0.229	0.10-0.52	<.001
	N=290	N=389			
High education, % (n)	54.8 (159)	29.6 (115)	2.89	2.10-3.97	<.001
	N=288	N=382			
Poor near vision, % (n)	36.5 (105)	43.5 (166)	0.75	0.55-1.02	.07

[a]ICT: information and communication technologies.
