# Supplementary material for: Use of Information and Communication Technologies Among Older People With and Without Frailty: A Population-Based Survey
Source: J Med Internet Res. 2017 Feb 14;19(2):e29. doi: 10.2196/jmir.5507 (PMC5331186; doi:10.2196/jmir.5507)
Supplement: Multimedia Appendix 5 [file jmir_v19i2e29_app5.pdf]

Multimedia Appendix 5. Attitude predictors of the use of advanced mobile information and communication technologies (ICT) in the last 12 months among Finnish seniors aged 65+ years in univariate regression. “I don’t know” answers counted as negative.

Attitude predictors	Mobile ICT[a] user[b] % (n)	Mobile ICT nonuser[b] % (n)	Odds ratio	95% CI	N	*P*
	N=286	N=376				
Interested in using ICT	67.8 (194)	30.3 (114)	4.85	3.48-6.76	662	<.001
	N=278	N=369				
Believes others would think positively of use (SI[b])	26.6 (74)	16.3 (64)	1.73	1.18-2.52	647	.005
	N=285	N=374				
Has privacy concerns	40.0 (114)	38.2 (143)	1.08	0.79-1.48	659	.65
**Considers mobile ICT**						
	N=282	N=371				
Useful (PE[c])	64.9 (183)	24.8 (92)	5.61	3.99-7.87	653	<.001
	N=285	N=371				
Easy to use (EE[d])	47.0 (134)	13.2 (49)	5.83	3.99-8.53	656	<.001
	N=277	N=365				
Too expensive	19.1 (53)	19.5 (71)	0.98	0.66-1.46	642	.98

[a]ICT: information and communication technologies.

[b]SI: social influence.

[c]PE: performance expectancy.

[d]EE: effort expectancy.
